# Supplementary material for: The Two-Component System 09 Regulates Pneumococcal Carbohydrate Metabolism and Capsule Expression
Source: Microorganisms. 2021 Feb 24;9(3):468. doi: 10.3390/microorganisms9030468 (PMC7996280; doi:10.3390/microorganisms9030468)
Supplement: Supplementary file 1 [file microorganisms-09-00468-s001.zip › TCS09 Supplement R1/20210202_FV_Supplement_TCS09_Manuscript_R1.pdf]

**Table S2.** Inline barcode sequences.

| BC ID | Barcode sequence 5′-3′ | Oligonucleotide sequence |
|---|---|---|
| L01 | CCAAGTCG | ACCAAGTCGAGATCGGAAGAGCGTCGTGTA |
| L02 | GCAGCCAC | AGCAGCCACAGATCGGAAGAGCGTCGTGTA |
| L03 | GTAACTGC | AGTAACTGCAGATCGGAAGAGCGTCGTGTA |
| L04 | TCATCGTG | ATCATCGTGAGATCGGAAGAGCGTCGTGTA |
| L05 | TTACCACG | ATTACCACGAGATCGGAAGAGCGTCGTGTA |
| L06 | AGAATTAT | AAGAATTATAGATCGGAAGAGCGTCGTGTA |
| L07 | GGCCCAAG | AGGCCCAAGAGATCGGAAGAGCGTCGTGTA |
| L08 | TACAACAT | ATACAACATAGATCGGAAGAGCGTCGTGTA |
| L09 | TGAACCAG | ATGAACCAGAGATCGGAAGAGCGTCGTGTA |
| L10 | ATATGGAC | AATATGGACAGATCGGAAGAGCGTCGTGTA |
| L11 | CAACTCGC | ACAACTCGCAGATCGGAAGAGCGTCGTGTA |
| L12 | CGGAGGGC | ACGGAGGGCAGATCGGAAGAGCGTCGTGTA |
| L13 | ACATTATT | AACATTATTAGATCGGAAGAGCGTCGTGTA |
| L14 | ATCACTTG | AATCACTTGAGATCGGAAGAGCGTCGTGTA |
| L15 | CCCGTCTT | ACCCGTCTTAGATCGGAAGAGCGTCGTGTA |
| L16 | CTCGGTAC | ACTCGGTACAGATCGGAAGAGCGTCGTGTA |
| L17 | GTCTGGCG | AGTCTGGCGAGATCGGAAGAGCGTCGTGTA |
| L18 | TCCCGCGG | ATCCCGCGGAGATCGGAAGAGCGTCGTGTA |
| L19 | CGGCACTT | ACGGCACTTAGATCGGAAGAGCGTCGTGTA |
| L20 | GAGATTGT | AGAGATTGTAGATCGGAAGAGCGTCGTGTA |
| L21 | TACAGATG | ATACAGATGAGATCGGAAGAGCGTCGTGTA |
| L22 | TGGGAGAC | ATGGGAGACAGATCGGAAGAGCGTCGTGTA |
| L23 | CCCTACAG | ACCCTACAGAGATCGGAAGAGCGTCGTGTA |
| L24 | CTCTAACT | ACTCTAACTAGATCGGAAGAGCGTCGTGTA |
| L25 | AAGTGTTG | AAAGTGTTGAGATCGGAAGAGCGTCGTGTA |
| L26 | GAGCCATC | AGAGCCATCAGATCGGAAGAGCGTCGTGTA |
| L27 | GGTCCTCT | AGGTCCTCTAGATCGGAAGAGCGTCGTGTA |
| L28 | TACCGGCC | ATACCGGCCAGATCGGAAGAGCGTCGTGTA |
| L29 | CCCTCGGC | ACCCTCGGCAGATCGGAAGAGCGTCGTGTA |
| L30 | CTGGATCG | ACTGGATCGAGATCGGAAGAGCGTCGTGTA |
| L31 | TTTCTAAC | ATTTCTAACAGATCGGAAGAGCGTCGTGTA |
| L32 | CCGGTACC | ACCGGTACCAGATCGGAAGAGCGTCGTGTA |

**Table S3.** Primers used for RNA preparation for RNA-sequencing.

| Index code | Barcode sequence 5′-3′ | Primer sequence 5′→3′ |
|---|---|---|
| AR2 | | TACACGACGCTCTTCCGAT |
| 3Tr3 | | iCiGiCCAGACGTGTGCTCTTCCGATCTrGrGrG |
| P5 | | AATGATACGGCGACCACCGAGATCTACACTCTTTC CCTACACGACGCTCTTCCGATCT |
| X01 | AAGTAGAG | CAAGCAGAAGACGGCATACGAGATAAGTAGAGGT GACTGGAGTTCAGACGTGTGCTCTTCCGATCT |

*iC = iso-dC; iG = iso-dG; rG = ribonucleotide G*

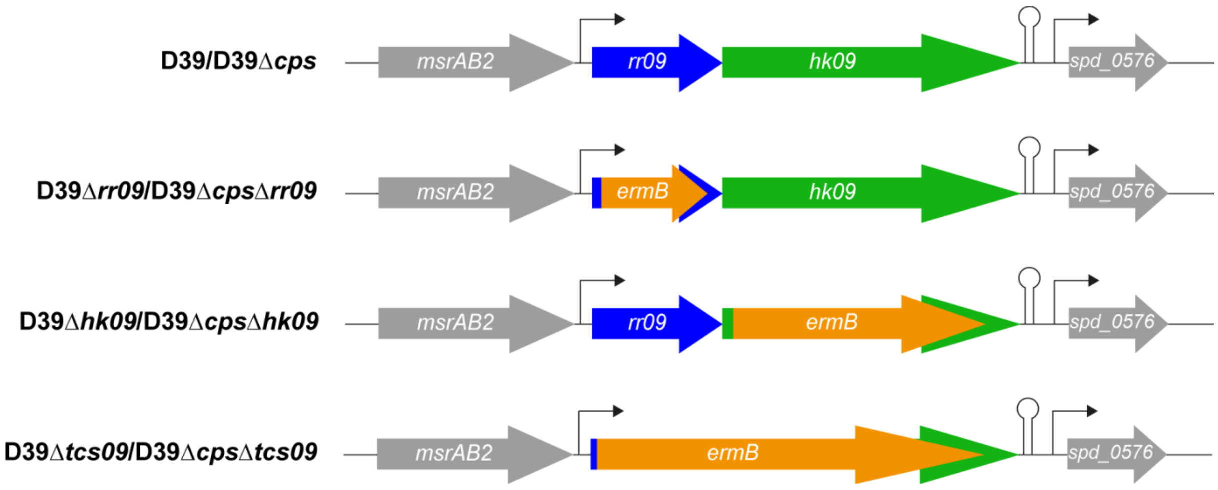


**Figure S1:** Genomic organization of the *tcs09* gene cluster in *S. pneumoniae* D39 wild-type and isogenic *tcs09*-mutants. The *rr09* gene is shown in blue and the *hk09* gene in green within its operon organization, the genes upstream and downstream of *rr09* and *hk09* are shown in grey and the inserted *ermB*- cassette for *tcs09* deletions is indicated by orange. The large and filled arrows represent their relative gene size and orientation in the genome. Transcriptional start sites are indicated with a thin arrow and terminators with lollypops.

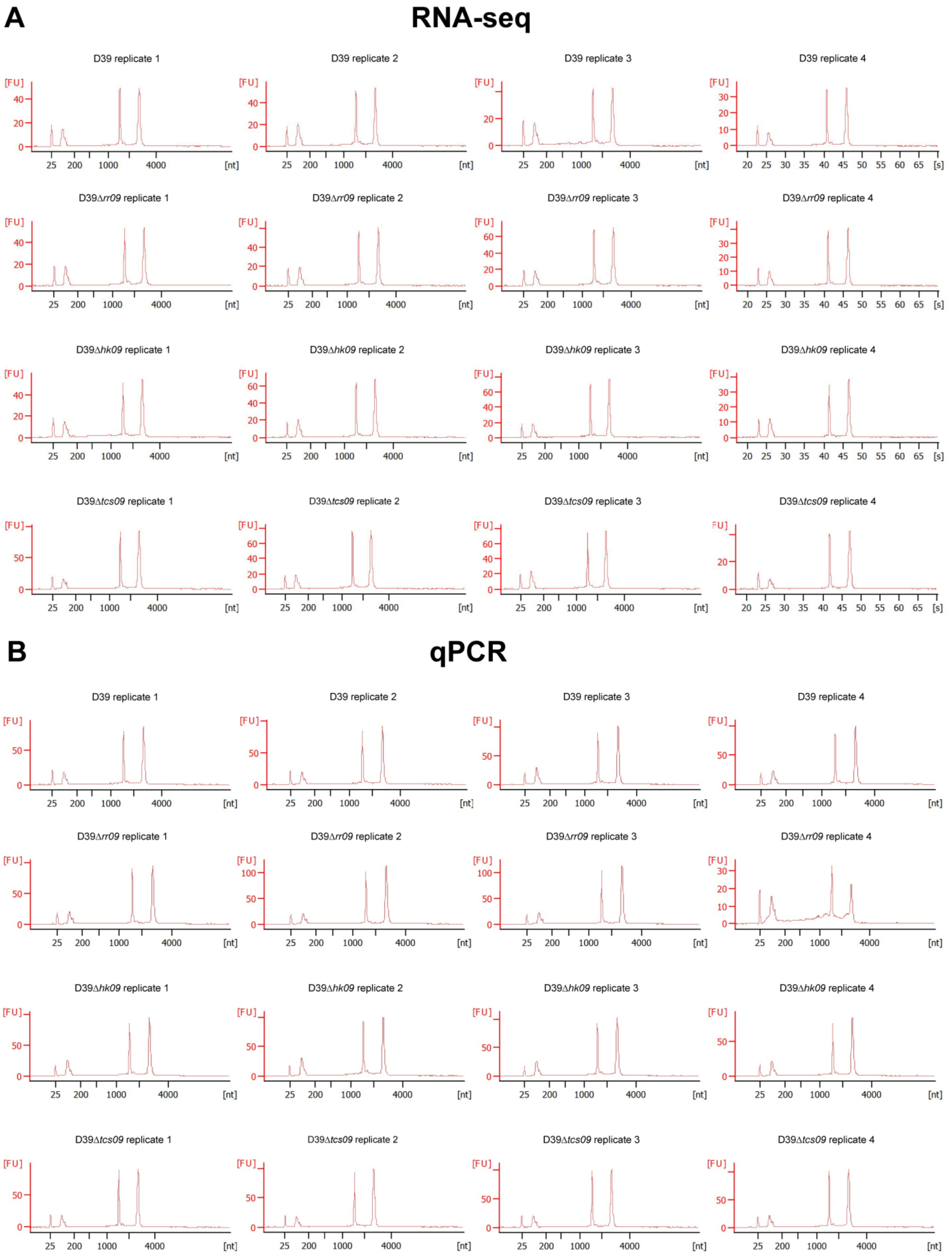


**Figure S2.** RNA integrity check with the Agilent Bioanalyzer. Shown are the electrophoretic representations of the individual RNA samples used for (**A**) RNA-seq and (**B**) qPCR. The curves consist of added marker and the individual ribosomal RNAs, which are used to calculate the RNA quality.

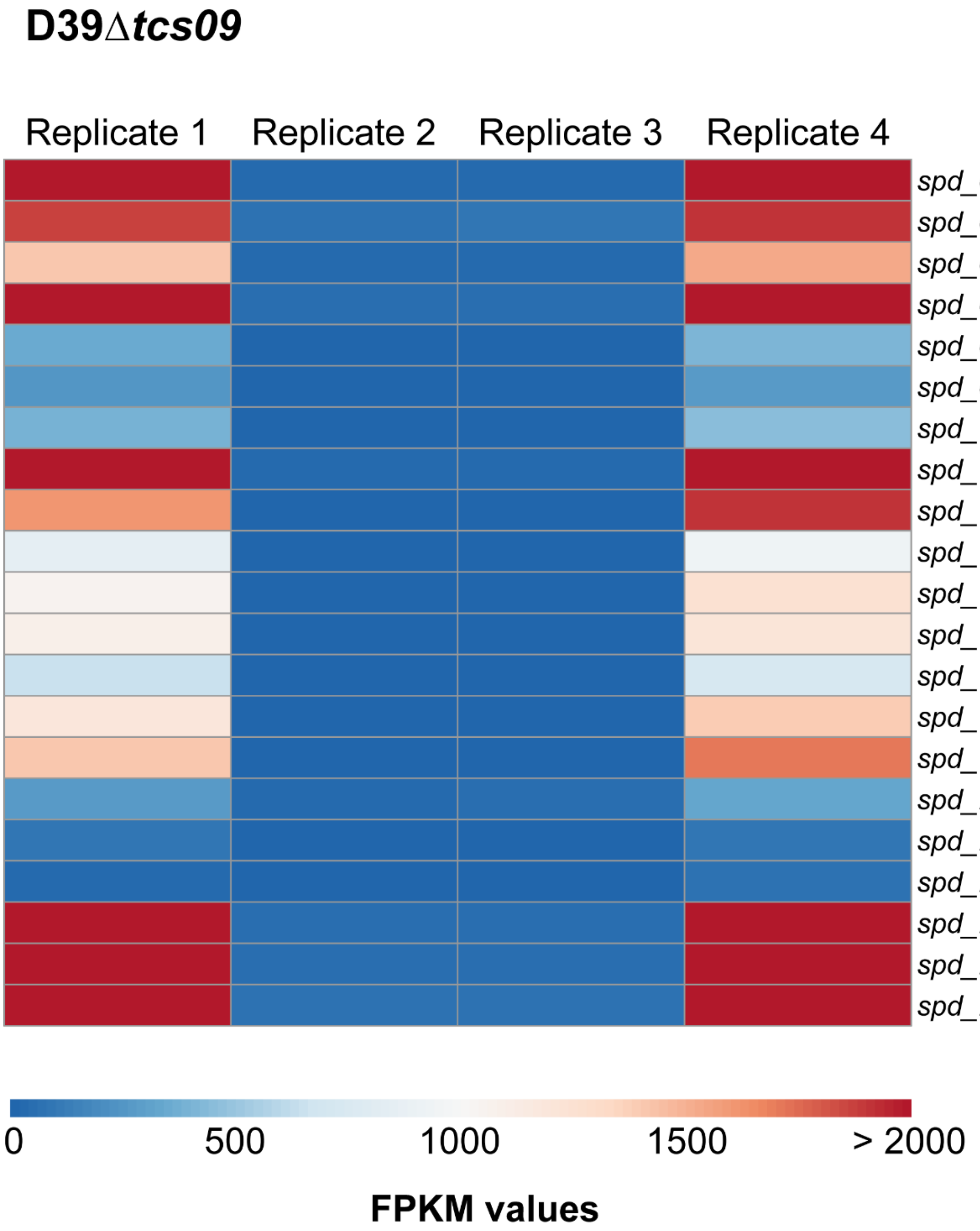


**Figure S3.** Expression profile of competence genes in D39Δ*tcs09*. Heat map showing FPKM values of competence genes in individual replicates of D39Δ*tcs09*. Red boxes show high values in the replicates, blue boxes low values.

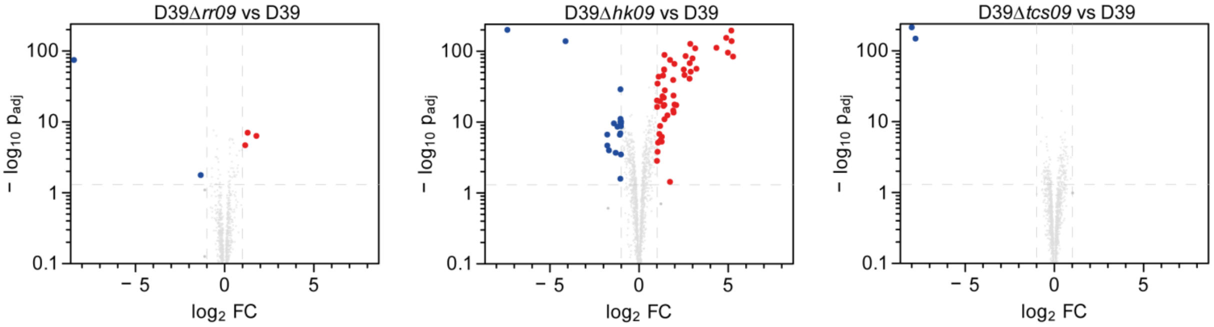


**Figure S4.** Volcano plots of the differentially expressed genes of D39Δ*rr09*, D39Δ*hk09* and D39Δ*tcs09* identified by RNA-seq. Histograms represent the two-dimensional distribution of identified genes by fold change and p-value. Genes with a $-\log_{10}$ $p_{adj}$-value ≥ 1.3 and a $\log_2$ fold change ≥ 1 or ≤ -1 were set significant. Significantly downregulated genes are shown in blue and significantly upregulated genes in red.

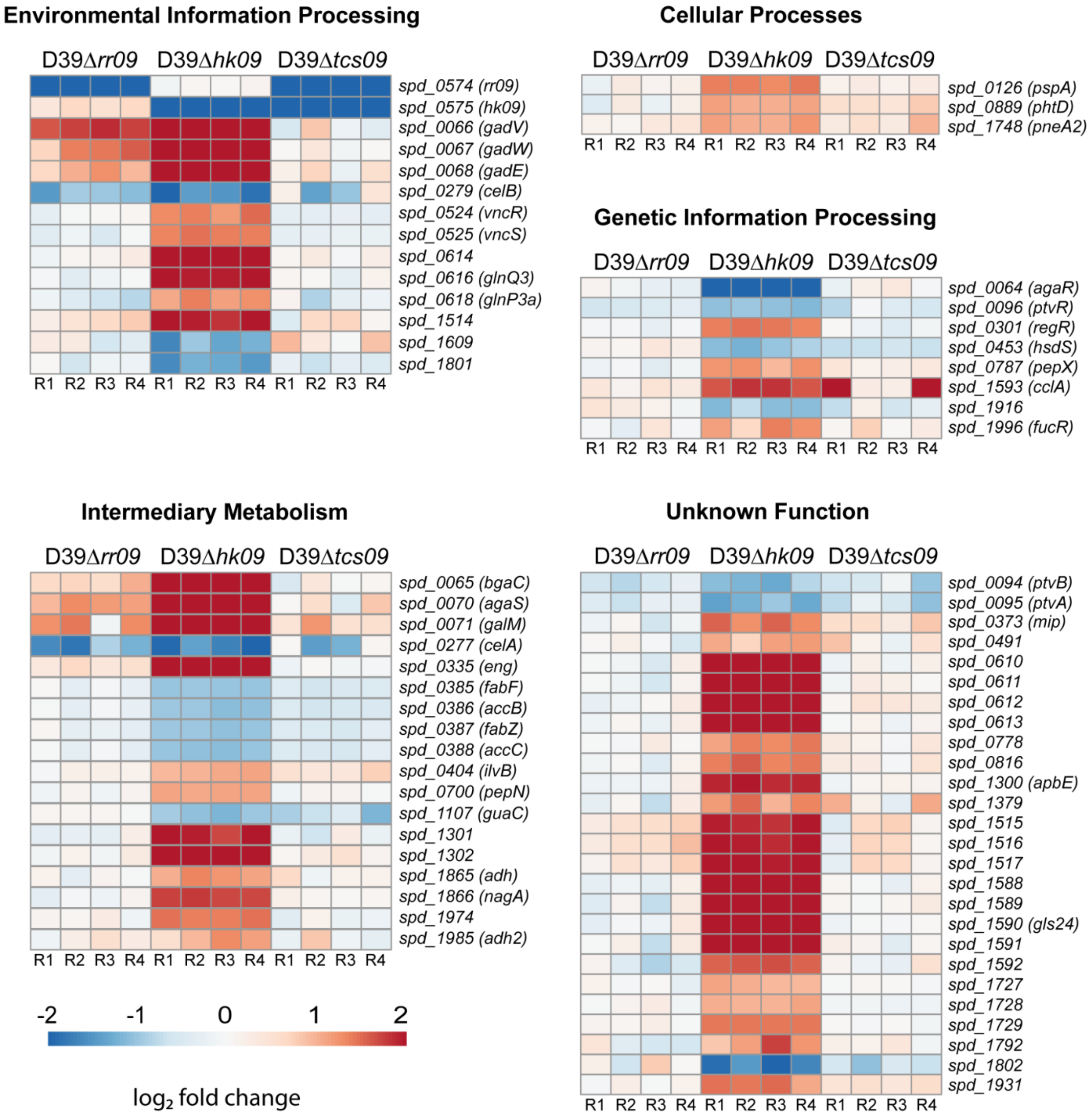


**Figure S5.** Expression profiles of 67 significantly regulated genes in D39 and *tcs09*-mutants. Heat maps showing the $\log_2$ fold changes in individual replicates of D39Δ*rr09*, D39Δ*hk09* and D39Δ*tcs09*. Red boxes show upregulation, whereas blue boxes show downregulation in the corresponding replicate.

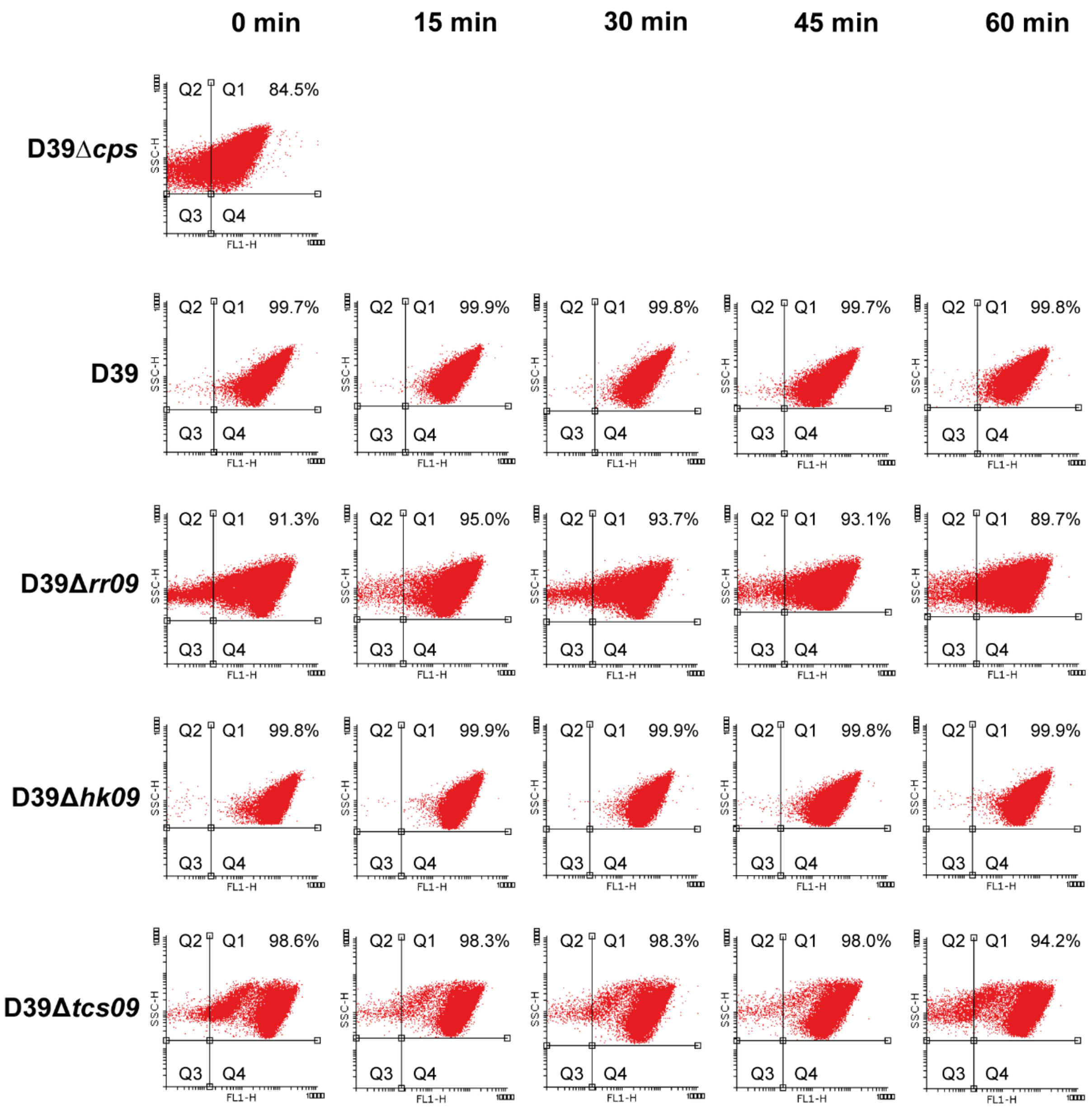


**Figure S6.** Analysis of capsule polysaccharide expression in flow cytometry. 2 x $10^8$ bacteria of the parental strains D39 and their isogenic Δ*rr09*-, Δ*hk09*- and Δ*tcs09*-mutants were incubated up to 60 min in PBS and afterwards capsule expression was analyzed with anti-serotype 2 and secondary Alexa conjugated antibody as described. As a control, 2 x $10^8$ bacteria of the nonencapsulated D39Δ*cps* was used in the analysis. Shown are the scatter plots of one representative measurement per time point. The percentages indicate the proportion of fluorescent cells within the selection of all cells in Q1. Q1-4: quadrant 1-4, SSC-H: side scatter height
